# Ultrasound and MRI Correlations with Pathological Findings in Fibrolipomatous Hamartoma of Peripheral Nerves

**DOI:** 10.2174/0115734056375869250728075232

**Published:** 2025-08-04

**Authors:** Kezhen Qin, Hengtao Qi, Yeting Wang, Wen Chen, Tiezheng Wang, Liyuan Cui, Huawei Zhang

**Affiliations:** 1 Department of Ultrasound, Shandong Provincial Hospital Affiliated to Shandong First Medical University, Jinan, Shandong, 250021, China

**Keywords:** Ultrasonography, Magnetic resonance imaging, Fibrolipomatous hamartoma, Peripheral nerve, Cable-like appearances, Neuropathic symptoms

## Abstract

**Introduction:**

The aim of this study was to evaluate the correlation between ultrasonography, magnetic resonance imaging, and pathology with Fibrolipomatous Hamartoma (FLH) of the peripheral nerve.

**Methods:**

Sixteen patients who underwent surgical treatment for FLH of the peripheral nerve were included in the study. Ultrasound examination and Magnetic Resonance Imaging (MRI) were used to display the detailed anatomical structure of the region well enough to detect FLH. The features presented based on the results of ultrasound examination and magnetic resonance imaging were recorded.

**Results:**

The involved peripheral nerve showed expansive growth in ultrasonography and MRI. The hyperechoic fat tissue and hypoechoic nerve fibers alternated with one another. In ultrasonography and MRI, the peripheral nerve exhibited a lotus-like appearance in the transverse plane, and a cable-like appearance in the longitudinal plane, while there was no blood flow signal in the nerve.

**Discussion:**

The imaging features of FLH, including the characteristic lotus-like and cable-like appearances, align closely with pathological findings, underscoring the diagnostic value of ultrasonography and MRI. These non-invasive techniques facilitate differentiation from other peripheral nerve pathologies, such as carpal tunnel syndrome or hemangioma. Limitations include the retrospective design, small MRI subgroup, and lack of long-term follow-up. Future multicenter studies with larger cohorts are recommended to validate these findings.

**Conclusion:**

Ultrasonography and MRI may be valuable in the diagnosis of FLH of the peripheral nerve.

## 1 INTRODUCTION

Fibrolipomatous Hamartoma (FLH) is an uncommon disorder that stems from the overgrowth of mature adipose tissue and fibroblasts within the epineurium, giving rise to fibrofatty infiltration and the expansion of a peripheral nerve. The median nerve is the most commonly affected peripheral nerve, accounting for approximately 60% - 80% of cases [1], while involvement of other peripheral nerves of the upper extremity is reported less frequently.

FLH patients often present in their third or fourth decade of life, frequently reporting a long history of painful or slightly painful swelling in the distal forearm, which may be associated with mild to severe neuropathic symptoms. Patients with median nerve involvement commonly exhibit carpal tunnel syndrome [2, 3]. Previously, the diagnosis of FLH was established through exploratory surgical procedures and biopsy of the affected area. Imaging techniques, such as ultrasonography and MRI, have become essential tools for accurate diagnosis and preoperative planning. The manifestation of FLH on magnetic resonance imaging has been thoroughly detailed in numerous case reports [4-6]. The magnetic resonance imaging characteristics shared by all instances of FLH have been examined and regarded as diagnostically specific. Nevertheless, the variety of morphological traits of FLH on ultrasonic imaging has been systematically investigated. In the present study, the aim was to evaluate the correlation between ultrasonography, magnetic resonance imaging, and pathology with FLH of the peripheral nerve.

## 2 MATERIALS AND METHODS

A retrospective analysis was conducted on sixteen cases of peripheral nerve FLH at our hospital. The patients (10 males and 6 females), aged between 5 and 41 years (with a mean age of 21.8 years), were treated from November 2013 to September 2024. Patients were included if they met the following criteria: (1) clinical suspicion of FLH confirmed by postoperative histopathology and (2) availability of preoperative ultrasonography and MRI data. Exclusion criteria were: (1) concurrent peripheral nerve tumors (*e.g.*, schwannoma, neurofibroma) and (2) incomplete imaging or pathological data. All patients presented with unilateral involvement; among them, eleven patients had macrodactyly. Seven patients had numbness in the thumb, forefinger, and the radial side of the middle finger. One patient had numbness on the lateral aspect of the foot’s dorsum. Sixteen cases were examined through ultrasonic scanning, while five cases underwent MRI. All of the sixteen patients received surgical treatment. The surgically resected specimens were fixed in formalin and embedded in paraffin, followed by Hematoxylin and Eosin (H&E) staining. The evaluation criteria for adipose tissue infiltration included the proportion and distribution of adipocytes under the microscope, while the evaluation criteria for fibrous tissue infiltration included the density of fibrous tissue and the thickening of nerve bundles. The study was conducted in accordance with the Declaration of Helsinki (as revised in 2013) and was approved by the Ethics Committee of Shandong Provincial Hospital affiliated with Shandong First Medical University (approval No. MR-37-23-023994). Informed consent was obtained from all participants. Ultrasound examinations were performed using a Philips IU22, Philips EPIQ 5(manufactured by Philips Healthcare), and a GE Vivid7 Dimension system (manufactured by GE Healthcare in Holten, Norway), along with a 12MHz or 14MHz broadband linear array transducer. All images were acquired with the patient in a relaxed state to minimize the impact of probe pressure on nerve morphology. The affected peripheral nerves were scrutinized, commencing from the proximal part and extending to the distal portion. The location, width, echogenic changes, as well as the adjacent anatomical structures encircling the nerve fascicles, were observed. The measurement site was the largest region of the affected nerve. In the transverse plane images, the nerve margins were manually traced to ensure clear visualization of the nerve's cross-section. Each case was measured at least three times, and the average value was taken to reduce errors. The corresponding region of the contralateral limb was also measured for comparison. All ultrasound examinations were performed using a sonographer with 17 years of experience in peripheral nerve ultrasonography. To evaluate the consistency of the measurements, another expert with 20 years of experience in peripheral nerve ultrasonography independently measured the cross-sectional areas in the ultrasonic images. Both physicians were blinded to the MRI results. The measurements from the two physicians were assessed using the Intraclass Correlation Coefficient (ICC) to determine consistency. Furthermore, a 1.5T magnetic resonance imaging scanner (MAGNETOM Avanto 1.5T, Siemens, Erlangen, Germany) equipped with a four-channel phased-array wrist coil was utilized to examine seven patients. The typical examination protocol comprised the subsequent sequences: Coronal T2-weighted Turbo Spin Echo (TSE), Coronal T2-weighted turbo spin echo with fat suppression (coronal T2 FS), coronal proton density-weighted turbo spin echo with fat suppression (coronal PD FS), sagittal proton density-weighted turbo spin echo (sagittal PD), and axial T1-weighted turbo spin echo (axial T1). The MRI parameters were as follows: slice thickness was 3 mm, the field of view was 160 mm × 160 mm, and no contrast agent was used. All peripheral nerve examinations were performed by a veteran technician with 18 years of expertise in musculoskeletal magnetic resonance imaging. All peripheral nerve images were independently assessed by senior radiologists with 21 years of experience in musculoskeletal imaging. The assessors were blinded to the MRI and color Doppler ultrasonography findings.

All statistical analyses were performed via SPSS (IBM SPSS Statistics, version 25.0; IBM Corporation, Armonk, NY, USA). Categorical variables were represented by numbers and percentages, and the differences between groups were examined using the chi-square test. Measurement data that conformed to a normal distribution were expressed as x-±s, and the differences between groups were analyzed by the independent-samples t-test. The effect size (Cohen's d) was calculated to assess the magnitude of the differences. Measurement data that did not conform to a normal distribution were presented as median (interquartile range), and the differences between groups were tested via the Mann-Whitney U test. The Intraclass Correlation Coefficient (ICC) was used to assess the consistency of the two physicians in measuring the aforementioned various measurement indices. Specifically, ICC < 0.4 was regarded as poor consistency, 0.4 ≤ ICC ≤ 0.75 was considered as moderate consistency, and ICC > 0.75 was deemed as good consistency.

## 3 RESULTS

**Fig. (3):**
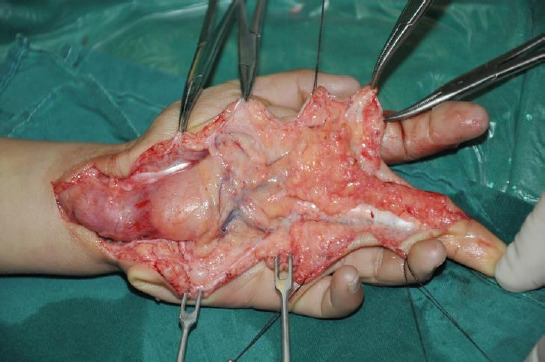
The enlarged peripheral nerves involved during the surgery and infiltrated with adipose tissue.

As ultrasonic and MRI images Figs. (**1** and **2**) show, both ultrasonic and MRI images distinctly demonstrate nerve fascicles as well as peripheral soft-tissue structures. The involved peripheral nerve showed expansive growth. The ultrasonic image revealed an expansive growth of the involved peripheral nerve, displaying a mixed echo mass characterized by alternating hyperechoic fat tissue and hypoechoic nerve fibers. In the transverse plane, the nerve exhibited a lotus-like appearance, while in the longitudinal plane, it appeared cable-like. Notably, no blood flow signal was detected within the nerve. On T1-weighted images, the high-signal adipose tissue and low-signal nerve fibers are interlaced, while the T2-weighted fat-suppressed sequences further clearly demonstrate the contrast between the nerve fibers and surrounding tissues. The lesion typically shows no significant enhancement, consistent with the pathological findings of fat infiltration and rare blood vessels.. At surgery, all peripheral nerve involvement was significantly enlarged and infiltrated by yellow adipose tissue (Fig. **3**). There was a clear demarcation between the anatomy of the peripheral nerve and the surrounding tissue.

The inter-observer agreement between the two ultrasound physicians for cross-sectional area measurements was evaluated using the Intraclass Correlation Coefficient (ICC), which showed excellent agreement (ICC = 0.92, 95% CI: 0.85–0.96). This indicates a high level of consistency between the two physicians in measuring cross-sectional areas. The mean cross-sectional area of FLH in the wrist joint or ankle joint at ultrasonography was 0.81 ± 0.22 cm^2^ in 16 patients, and the mean diameter of the contralateral upper extremity was 0.10 ± 0.02 cm^2^, which shows a significant difference (*t* = 12.83, *p* < 0.01). The effect size (Cohen's d) was 4.55, indicating a very large effect size for the difference in cross-sectional area between FLH and the contralateral normal nerve fascicles.

## 4 DISCUSSION

FLH is a rare condition, and its precise occurrence rate has not been thoroughly detailed in the existing literature. It usually emerges during childhood or in the early stages of adulthood. When FLH occurs in the carpal tunnel, the increased pressure within the carpal tunnel can lead to compression of the median nerve, potentially causing carpal tunnel syndrome in children [7]. It has also been referred to as fibrolipomatous nerve hypertrophy, lipofibroma, fibrofatty proliferation, neural fibrolipoma, or neurolipoma. In 2002, the World Health Organization classified it as a lipomatosis of the nerve [8].

At present, the etiology of FLH remains unclear [9, 10]. The proposed mechanisms in the literature include the congenital dysplasia theory, the imbalance theory of nerve-fat-fiber tissue interaction, the acquired factor-induced theory, and so on. Warren RB reported a case of precalcaneal congenital FLH, suggesting the possibility of its congenital origin [11]. Marom EM believes that in FLH, adipose tissue hyperplasia and interweaving with fibrous tissue infiltrate the nerve bundles, resulting in damage to the nerve structure and function [12]. There is also a view that FLH can be acquired later in life and is related to factors such as trauma, nerve irritation, and inflammation. Guthikonda proposed that chronic microtrauma to the median nerve caused by the wrist ligament could lead to the development of FLH [13]. In this study, all patients with FLH exhibited clinical syndromes since childhood, consistent with a congenital disease.

**Fig. (4):**
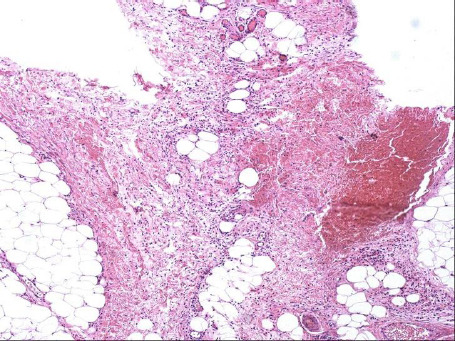
Pathological sections of fibrolipomatous hamartoma.

This study highlights the diagnostic value of ultrasonography and MRI in identifying FLH of peripheral nerves. Both imaging modalities demonstrated promising diagnostic utility in detecting the characteristic features of the lesion, which were consistent with pathological findings. The US and MRI are the quickest and most reliable ways for establishing the correct diagnosis of FLH [2]. In FLH, the perineural and epineural regions of the affected peripheral nerve histologically display infiltration by mature adipose tissue combined with fibrous tissue. These infiltrates penetrate and isolate individual nerve bundles (Fig. **4**). Additionally, atrophy accompanied by concentric perineural fibrosis, leading to the thickening of nerve fascicles, can be observed. At ultrasonography and MR imaging of the present patients, the hyperechoic fat tissue and hypoechoic nerve fibers alternated with each other. The peripheral nerve had a lotus-like appearance in the transverse plane, and a cable-like appearance in the longitudinal plane, which was consistent with the findings of pathological sections. There were rare blood vessels in pathological sections of FLH, which were consistent with the color Doppler ultrasonography appearances. MRI offers unique advantages in diagnosing FLH, particularly in displaying the interlaced distribution of nerve fibers and adipose tissue. On T1-weighted images, adipose tissue appears as high signal intensity, while nerve fibers show low signal intensity, which is highly consistent with pathological findings. Additionally, coronal and axial MRI images clearly demonstrate the lotus-like appearance of the nerve, further supporting its diagnostic value [4-6]. Compared to MRI, ultrasound offers several advantages, including the ability to provide real-time imaging, being time-efficient and inexpensive, having zero radiation exposure, and possessing high resolution for superficial soft tissues [5, 14, 15].

Patients with FLH typically present with a slowly growing soft-tissue mass; therefore, FLH is often identified with hemangioma in clinical practice. Typical ultrasonographic patterns include heterogeneous echogenicity, ill-defined margins, solid and cystic in composition, and hypervascularity. However, good compressibility, echogenic rim, and the presence of phleboliths have a very high positive predictive value for hemangioma [16]. Besides, it is important to distinguish FLH from Carpal Tunnel Syndrome (CTS) in clinical practice, as both conditions may present with median nerve enlargement. In CTS, the median nerve typically shows hypoechoic features and is enlarged at the carpal tunnel inlet, with a noticeable decrease in diameter within the carpal tunnel, often accompanied by bowing of the flexor retinaculum. In contrast, FLH presents with a characteristic mixed echo pattern, featuring alternating hyperechoic fat tissue and hypoechoic nerve fibers, and the nerve maintains its enlarged diameter throughout its course without the typical bowing of the flexor retinaculum observed in CTS. These distinct ultrasonic features aid in the accurate diagnosis of FLH and help avoid misdiagnosis as CTS [17, 18].

Concerning the management of FLH, complete excision of the involved nerve is not advocated in every instance. This is because it may have adverse impacts on motor and sensory functions and may also lead to postoperative neurogenic pain. Furthermore, in this invasive process, it can be challenging to attain the ideal resection margin. Thus, conservative treatment along with decompression of the affected nerves is typically employed [4]. At present, Carpal Tunnel Release (CTR) is the primary treatment method for carpal tunnel syndrome. It has been confirmed that it is helpful for many patients with carpal tunnel symptoms and can completely resolve the symptoms [19, 20].

However, this study has some limitations. The retrospective design, small sample size (particularly for the MRI subgroup, n = 5), and single-center data may limit the generalizability of the results. Additionally, the study only observed the correlation between preoperative ultrasound and MRI findings and pathology in patients, lacking long-term follow-up of postoperative patients. As a result, it is impossible to clarify the value of ultrasound and MRI in evaluating disease recurrence, treatment efficacy, and other aspects. While the findings are promising, larger, prospective, and multicenter studies are needed to validate the diagnostic accuracy of ultrasonography and MRI in FLH. Future research could consider expanding the sample size, incorporating multi-center collaborations, and adding long-term postoperative follow-up to more comprehensively evaluate the role of ultrasound and MRI in the diagnosis and treatment of FLH.

## CONCLUSION

In conclusion, ultrasonography and MRI represent valuable diagnostic modalities for the evaluation of FLH of peripheral nerves, demonstrating promising diagnostic utility. The characteristic imaging features exhibit strong correlation with pathological findings, reinforcing their diagnostic reliability. These non-invasive imaging techniques offer dual clinical benefits: they not only facilitate accurate preoperative diagnosis but also serve as effective tools for differentiating FLH from other peripheral nerve pathologies.

## Figures and Tables

**Fig. (1):**
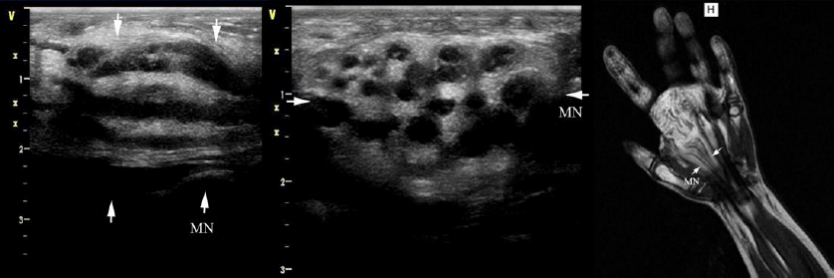
Ultrasonic and MRI images of fibrolipomatous hamartoma of the enlarged median nerve.

**Fig. (2):**
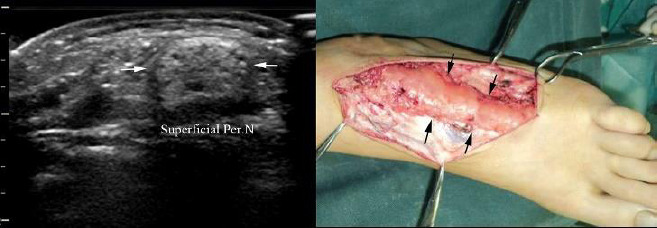
Ultrasonic images and intraoperative conditions of the enlarged superficial peroneal nerve.

## Data Availability

The data and supportive information are available within the article.

## LIST OF ABBREVIATIONS

MRI: Magnetic Resonance Imaging
FLH: Fibrolipomatous Hamartoma
H&E: Hematoxylin and Eosin
ICC: Intraclass Correlation Coefficient

## STANDARDS OF REPORTING

STROBE guidelines were followed.
